# Validating a Reading Comprehension Assessment for College Students: Preliminary Findings

**DOI:** 10.1007/s10936-025-10144-6

**Published:** 2025-05-03

**Authors:** Sarah E. Carlson, Virginia Clinton-Lisell, Terrill Taylor, Heather Ness-Maddox, Amanda Dahl, Mark L. Davison, Ben Seipel

**Affiliations:** 1 Georgia State University, Atlanta, USA; 2 Educational Foundations and Research, University of North Dakota, 231 Centennial Ave., Grand Forks, ND 58202 USA; 3 University of Maryland, College Park, USA; 4 Middle State Georgia University, Macon, USA; 5 Michigan State University, East Lansing, USA; 6 University of Minnesota, Saint Paul, USA; 7 California State University, Chico, USA

**Keywords:** MOCCA-college, Reading comprehension, Inferences, Assessment, College students

## Abstract

The purpose of this study was to validate a novel reading comprehension assessment for college students named MOCCA-College. A random sample of college students (*N* = 63, average age of 22.5) were recruited from various education programs (e.g., first-year courses, TRIO, SONA) and completed MOCCA-College Online and were later recruited to complete face-to-face think-aloud and recall tasks, as well as standardized assessments such as the Nelson-Denny Reading Test (NDRT) and the Test of Word Reading Efficiency (TOWRE-2). Based on the think-aloud findings, correct answers on MOCCA-College were associated with meaningful connections to background knowledge. Incorrect answers were associated with irrelevant connections to background knowledge that are not helpful for comprehension. Moreover, efficiency on MOCCA-College (seconds per correct answer) demonstrated criterion validity based on the NDRT and TOWRE-2. Future research and analyses may examine assessment development, particularly for identifying nuanced individual differences in college readers’ comprehension.

Reading comprehension skills are critical for success in college (Desa et al., 2020; Gregory & Bean, 2021; Howard et al., 2018). Therefore, postsecondary institutions provide reading support for students through various remedial and general instruction, as well as through tutoring resources. However, there is a lack of understanding of the specific student needs required for improving reading comprehension skills above and beyond what is found from national standardized reading scores (e.g., ACT, SAT). Thus, there has been recent and continuing pressure to develop reliable and valid measures to identify, diagnose, and address the reading comprehension needs of students entering postsecondary institutions (Holschuh & Paulson, 2013; Koon & Petscher, 2016). Specifically, examining reading comprehension processing skills are especially important given that many college students struggle with these skills (Feller et al., 2020). To address this need, the MOCCA-College was developed (Seipel et al., 2023). The purpose of this study is to examine initial evidence on the construct and criterion validity of the MOCCA-College through examining its relation to a methodology in which its foundation was built from (i.e., the think-aloud method), as well as outcomes of reading comprehension used to identify the representation of a text built after reading (i.e., recall).

## Theoretical Background

Some college readers may have sufficient decoding skills, but difficulty understanding what they read, resulting in poor reading comprehension (Barth et al., 2015; Feller et al., 2020; Magliano et al., 2020; Spencer & Wagner, 2018). In general, describing comprehension independent of decoding, the *construction-integration model* provides a framework of the process of comprehension (Kintsch, 1988). During the construction, readers activate and integrate background knowledge at various levels relevant to the text, which is through a dynamic process during reading (Kintsch, 1998). A key assumption of the construction-integration model is that there are three levels of knowledge representation from the text that readers activate and integrate during reading: the surface structure, the textbase, and the situation model. The surface structure is the literal words and syntax of the text (i.e., where decoding is critical). The textbase is composed of the ideas of the text, known as propositions, which are connected together in a coherent manner through text-connecting inferences (Graesser et al., 1994). Finally, readers integrate relevant background knowledge with the textbase to develop a situation model (Kintsch, 2019). Inferences, in which the reader connects ideas within the text or between the text and background knowledge, are necessary for successful textbase and situation models (Kendeou, 2015). As the reader encounters new ideas, the situation model is updated to accommodate their understanding of the text. For instance, consider this sentence used as an example in Perfetti and Stafura (2015): “While Cathy was riding her bike in the park, dark clouds began to gather, and it started to storm.” To fully understand this sentence, a reader needs to update their mental representation of Cathy riding a bike in the park with dark clouds overhead to add the event of the storm. When reading the subsequent sentence, “The rain ruined her beautiful sweater,” not surprisingly, the reader anticipates the word “rain” because of the text-connecting inferences for which then, presumably, the situation model updated and inferred to include the word “storm” (which generally involves rain; Perfetti & Stafura, 2015).

The creation and updating (i.e.,“during” reading) of the situation model are where the processes of comprehension take place (Kendeou et al., 2014, 2016). One important process is the causally coherent inference in which the reader connects cause and effect ideas in the text and related background knowledge to maintain coherence (Trabasso & van den Broek, 1985). To make a causally coherent inference, readers connect ideas in the text to understand why these ideas occurred and what their effects may be in a manner that is not explicitly stated in the text (Trabasso & van den Broek, 1985). Consider this example from Thurlow and van den Broek (1997): “Toby wanted to get Chris a present for his birthday. He went to his piggy bank.” Skilled comprehenders easily and causally infer that Toby goes to his piggy bank to get money to buy Chris a present by integrating their background knowledge about piggy banks and gifts with the information presented in the text. Without this inference, there is no connection between the cause of the desire to purchase a present and the effect of getting the piggy bank because the reason for getting the piggy bank is not explicitly stated in the text (Millis & Graesser, 1994). Subsequently, without the inference the reader would not be able to develop a coherent situation model or mental representation of the sentences.

Struggling comprehenders do generate inferences to develop and maintain coherent mental representations of text (Feller et al., 2020). However, struggling comprehenders tend to make fewer causal inferences than their more-skilled peers (Magliano et al., 2020). Furthermore, struggling comprehenders tend to be more likely to engage in cognitive processes other than causal inferences (Denton et al., 2015). The two most common of these non-causal processes are paraphrases and elaborations (Carlson et al., 2014; McMaster et al., 2012). Paraphrases are reiterations or summarization of the text and lack connections to other ideas in the text or background knowledge. As such, paraphrases are constrained to the text. In contrast, elaborations are connections to background knowledge in manners that do not contribute to causal coherence such as invalid or inaccurate connections to background knowledge or tangential associations. Importantly, and for the purpose of this study and the development of MOCCA and MOCCA-College (see the *Designing MOCCA-College* section below), we use the term elaborations because researchers have used this term and similar terms (e.g., associations, explanations, knowledge-based inferences, lateral connections) interchangeably, while others see them as distinct processes. For example, Wolfe and Goldman (2005) characterize elaborations as self-explanations that explain why the text is related to oneself. These cognitive processes have been researched with children, prompting a need to examine if college students engage in similar processes.

### Designing MOCCA-College

Many postsecondary institutions support their students’ reading skills through coursework, workshops, centers, and tutoring (Scott-Clayton & Rodriguez, 2015; Tinto, 2012). However, without reliable and valid methods of identifying students who struggle with reading in different ways, and in particular developing coherent situation models or mental representations of text to support successful comprehension, it is difficult for institutions to determine which students need assistance as well as in assessing the effectiveness of services provided. To address these issues, MOCCA-College was developed. MOCCA-College would address these needs by functioning as a diagnostic tool for postsecondary students who struggle with reading comprehension processing skills. Furthermore, MOCCA-College could be used to evaluate the effectiveness of reading programs with formative information that could guide additional instructional support.

MOCCA-College was developed based on prior work developing a diagnostic reading comprehension measure for intermediate elementary school students, known as MOCCA^1^ (intermediate or Grades 3–5; Biancarosa et al., 2019; Carlson et al., 2014; Davison et al., 2018) throughout this manuscript to avoid confusion with the MOCCA-College (Seipel et al., 2023). Both MOCCA and MOCCA-College are computer administered, discourse-level maze tasks. Unlike other maze tasks in which a single word is deleted at every seventh word in a text, an entire sentence is deleted from short seven-sentence texts. This missing sentence is critical for informing a causal connection or inference. During test administration, readers are tasked with choosing one of three sentence options that best completes the missing sentence for the item. Like most assessments, there is one correctly keyed option. This is the option that allows for the text to be best completed in a causally coherent manner using a response type that is termed the causally coherent inference option. Unlike many other assessments, the incorrect options on MOCCA-I and College are designed to indicate two cognitive processes that readers often engage in when they struggle with comprehension: paraphrases and elaborations. The incorrect option of the paraphrase is a sentence restating a previous sentence in the text item. In contrast, the incorrect option of the elaboration involves using tangential background knowledge that is not necessary for a coherent mental representation of the text. These response types were developed based on data from prior think-aloud studies that indicate struggling comprehenders tend to generate more paraphrases or elaborations during reading than other more causally coherent driven processes (i.e., causal inferences; Carlson et al., 2014; McMaster et al., 2012). Research findings on MOCCA-I have indicated that it is reliable and valid both as an assessment of comprehension accuracy and as a diagnostic tool for identifying why readers may struggle with comprehension (i.e., engaging in excessive paraphrasing or elaborations; Biancarosa et al.; Carlson et al.; Davison et al.; Su & Davison, 2019).

## The Current Study

The purpose of the current study is to examine the construct and criterion validity of MOCCA-College. To address this purpose, a think-aloud task was used with a sample of participants who completed MOCCA-College. In a think aloud, readers are asked to verbalize their thoughts as they read a text aloud (Ericsson et al., 1993). This task provides rich data and is useful for illuminating the cognitive processes in which readers engage. From think-aloud studies, researchers know readers engage in a variety of processes while reading (e.g., Karlsson et al., 2018). Particularly relevant for this study are the two types of processes that struggling comprehenders have been shown to overly rely upon: paraphrases and irrelevant connections to background knowledge, or elaborations, which include a combination of elaborative inferences, personal associations, and explanations (Carlson et al., 2014; McMaster et al., 2012; Seipel et al., 2017). Struggling comprehenders tend to overuse these processes whereas readers with stronger comprehension skills are more likely to generate inferences in which ideas in the text are effectively connected to each other (text-based inferences) or ideas in the text connect to missing information that is related to the text and their background knowledge (causal inferences; Kraal et al., 2018). The response options for MOCCA-College are designed to reflect these processes (paraphrases, elaborations, causally coherent inferences). Thus, examining how think-aloud processes are associated with MOCCA-College response options can help to further validate the need for developing the assessment.

In addition to examining think-aloud processes, memory for text was examined using a recall task. Recall is used in reading comprehension studies to understand the representation of a text that is developed after reading is complete (e.g., Theide et al., 2010). This task is also commonly used in conjunction with think alouds to further understand how the processes used during reading impact the readers’ representation or product developed of the text after reading (e.g., van den Broek et al., 2001). We analyzed readers’ recall responses in two ways. First, memory was examined in terms of the overall number of ideas in the text that were recalled. This was examined because findings from previous research indicate the amount recalled from a text is positively correlated with reading skill (Cao & Kim, 2020). In this way, a positive correlation between the amount remembered and correct answers on MOCCA-College would provide validating evidence. Second, memory was examined based on the importance of the idea in the text for causal coherence. Ideas vary in how highly connected they are in terms of being causes and effects of other ideas in the text. Typically, skilled readers recall highly connected idea units more than idea units with fewer causal connections (Carlson et al., 2022; McMaster et al., 2012; van den Broek et al., 2009). This is because highly connected idea units are central to the structure of a text and therefore more important than other idea units (van den Broek et al., 1996). In addition, as comprehension improves through age and experience, readers become more likely to recall highly connected idea units compared to other idea units (Pavias et al., 2016; van den Broek et al., 2009; Yeari & Lev, 2021). This is because readers develop *sensitivity to structural centrality* and are more aware of what ideas are key to comprehending a text (van den Broek et al., 2012). Because MOCCA-College is intended to assess causally coherent inferencing skills, it is important to consider how accurate responses on MOCCA-College relate to recall of highly-connected idea units as well as other idea units in order to assess construct validity.

MOCCA-College has also been programmed to collect timing data; however, participants are encouraged to use as much time as needed to complete as many items as possible. This allows for both reading comprehension accuracy and efficiency (i.e., reading time for accuracy) to be examined. Accuracy is clearly relevant as college students need to understand texts to be successful academically. Efficiency is also relevant as it is an indication of automaticity (processing without conscious effort) of reading skills (Samuels & Flor, 1997). Because reading comprehension involves multiple cognitive processes including decoding, vocabulary retrieval, and inferences, at least some of the skills must be automatic to allow for enough cognitive capacity to engage in all of the processes needed (Samuels & Flor, 1997). Lower-level cognitive skills such as decoding become automatic as readers develop proficiency (Megherbi et al., 2018; Roembke et al., 2021). In addition, vocabulary retrieval (i.e., understanding the meanings of words in the text) may become an automatic process (Perfetti, 2010). Generating inferences has generally been considered a more effortful process than the other lower-level skills (once automatic), but there is evidence that some inferences are generated automatically once developed (Thurlow & van den Broek, 1997), particularly by skilled readers (van den Broek et al., 2006, 2015). In summary, reading efficiency is dependent upon automaticity of multiple reading skills and automaticity is a strong indication of skilled reading. For these reasons, reading efficiency has been noted as an important measure of global reading skills that needs attention separate from reading comprehension accuracy independent of reading time (Skinner et al., 2009; Su & Davison, 2019); and thus, this is an additional skill measured with MOCCA-College. In contrast, response time for incorrect responses and overall response time (regardless of accuracy) have not been found to be useful measures of reading skill (Su & Davison, 2019), and we will not examine this variable in the current study.

As explained above, MOCCA-College was developed to assess different aspects of reading comprehension (i.e., cognitive processing during reading), in particular, reading efficiency (i.e., accuracy and multiple reading skills such as inference generation) being an important component of MOCCA-College. Therefore, criterion validity is examined in the following study through established reading measures. Namely, the Nelson-Denny Reading Test (NDRT) subtests of comprehension and reading rate are used. Unlike MOCCA-College which has independent items, the NDRT is a traditional passage-based assessment with multiple-choice questions after each passage (Fishco, 2019). However, because both MOCCA-College and the NDRT are designed to assess reading comprehension skills, a positive correlation between the two would provide evidence of criterion validity. Because the NDRT is time limited, a negative correlation between the comprehension score and reading MOCCA-College efficiency (seconds per correct item) is also expected. The NDRT reading rate measure is indicative of reading speed without considering accuracy; therefore, a negative correlation between reading rate and MOCCA-College efficiency would be indicative of criterion validity. In addition, we use measures of decoding skill (i.e., the Test of Word Reading Efficiency-2; TOWRE-2) to assess aspects of criterion validity. MOCCA-College does not assess decoding, but decoding skills are necessary to complete MOCCA-College. Because the TOWRE-2 measures are timed, a negative correlation between them and MOCCA-College reading efficiency is expected.

Thus, three research questions guided the current study:

1. To what extent do MOCCA-College responses, think-aloud processes, and recall, correlate as a means to identify construct validity? To observe evidence of construct validity, we hypothesize that MOCCA-College causally coherent inference responses (accurate) would positively correlate with valid elaborative inferences from the think-aloud task, as well as the highly connected idea units recalled from the text. In addition, we hypothesize that MOCCA-College causally coherent inference responses would negatively correlate with invalid elaborative inferences and paraphrases from the think-aloud responses. To observe further evidence of construct validity, and because MOCCA-College was developed to identify not only accurate responses, but also trends in inaccurate responses, we hypothesize MOCCA-College elaboration responses would positively correlate with invalid elaborative inferences from the think-aloud task, and MOCCA-College paraphrase responses, would positively correlate with paraphrases from the think-aloud task.
2. When controlling for decoding skills, to what extent do MOCCA-College responses and efficiency correlate with the think-aloud processes as one last piece of evidence for construct validity? Construct validity evidence here would be similar to that in research question one, but regression analyses will allow for more thorough examination.
3. To what extent do MOCCA-College responses correlate with previously established reading measures as evidence of criterion validity? To observe evidence of criterion validity, we hypothesize MOCCA-College causally coherent inference responses would positively correlate with outcomes from the NDRT subtests of reading comprehension. In addition, evidence of criterion validity for efficiency, we hypothesize MOCCA-College efficiency scores (seconds per correct response) would negatively correlate with the NDRT subtest of reading rate because longer response times would indicate lower efficiency.

## Method

### Participants

A total of *N* = 998 undergraduate students completed the MOCCA-College Online test at locations throughout the United States (i.e., Western, Midwestern, Northeastern, and Southeastern regions). The demographics of the participants for the larger MOCCA-College Online study (*N* = 998) were average age 22.56 years (SD = 6.78 year) with the majority identify as women, 29% identifying as men, and 1% identifying genderfluid/transgender/gender nonconforming or did not report a gender identity. The majority of participants identified as White, with 6% identified as Asian, 13% as Black or African American, 17% as Latino/a/x, 2% identified as Native American/Alaska Native/Hawaiian/Pacific Islander, and 5% reported a different racial identity, multiple identities, or preferred not to share their racial/ethnic identity. Participants were recruited for initial participation in the MOCCA-College through first-year courses, TRIO programs, Educational Opportunity Programs, SONA point systems, and student list-serves.

All participants who completed the MOCCA-College online responded to a request to participate in a follow-up study on validating evidence from MOCCA-College and were further contacted to arrange individual sessions to participate in the think-aloud and recall tasks, as well as take the NDRT and TOWRE-2 assessments. Participants for the validation face-to-face study, and from data reported here, were undergraduate students (*N* = 63) from two institutions both located in the United States and differed potentially in terms of the demographics of the students who attend each (one Midwestern, and one Western institution). The average age of participants in the validation study was 21.40 years (SD = 5.06 years). In terms of gender identity, 18 (29%) reported identifying as men, 43 (68%) reported identifying as women, and 1 reported identifying as gender nonconforming (one participant did not report a gender identity). In terms of racial and ethnic identities, 12.7% reported being Latino/a/x, 3.2% reported being Asian, 4.8% reported being Black or African American, 1.6% reported being Native American, 79.4% reported being White, and 1.6% preferred not to report.

The differences in racial and ethnic identities between the larger MOCCA-College participants and those who participated in follow-up validity study are likely due to the nature of one of the institutions in which face-to-face data were collected being a predominantly white institution. The data that support the findings of this study are available from the corresponding author upon reasonable request. Descriptive statistics of participants’ MOCCA-College responses, efficiency, and other measures in this study are listed in Table 1. This is a subset of a sample previously reported (Authors, date) that is analyzed with additional measures through think-aloud data collection to address additional questions.

**Table 1:** Descriptive statistics of study measures

	Minimum	Maximum	Mean	SD	
MOCCA-College responses					
Number of causally-coherent inference responses (correct)	12	50	40.56	8.58	
Number of paraphrase responses (incorrect)	0	23	4.51	4.044	
Number of elaboration responses (incorrect)	0	18	4.37	4.26	
Seconds/Causally-coherent inferences responses	9.87	250.57	66.43	40.43	
Think-aloud responses					
Number of knowledge-based inferences	6	85	42.87	15.88	
Number of text-connecting inferences	0	28	10.27	6.20	
Number of paraphrases	0	37	9.54	9.49	
Number of elaborations	0	28	8.24	5.94	
Text recall					
Number of idea units recalled	9	48	31.24	9.16	
Number of highly-connected idea units recalled	1	17	10.65	3.22	
Number of other idea united recalled	5	32	20.49	6.89	
Nelson-Denny Reading Test (index scores)					
Reading rate	78	141	101.21	13.27	
Comprehension score	70	143	114.16	14.92	
Test of Word Reading Efficiency-2 (scale scores)					
Sight Word Efficiency	75	130	101.25	11.52	
Phonemic Decoding Efficiency	78	130	104.05	10.93	

*NotesN* = 63. TOWRE-2 scale scores have been normed to have a mean of 100 and standard deviation of 15 (Torgesen et al., 2012). NDRT index scores have also been normed to have a mean of 100 and standard deviation of 15 (Fishco, 2019)

## Think-Aloud and Recall Materials

There were four texts used for the think-aloud and recall portions of this study of which two were narrative and two were expository (adapted from van den Broek et al., 2006; see Table 2 for information about the texts using Coh-Metrix, a computerized text analysis program, Graesser et al., 2014; McNamara et al., 2014). The texts ranged from 14 to 19 sentences long and 183 to 202 words long. Texts with Flesch-Kincaid grade levels below college grade level (5.93–7.50) were selected to minimize potential decoding difficulties given the focus of the think-aloud and recall tasks (see below) were on examining how readers make meaningful connections with the text. In addition, these texts were selected to be similar to some of the MOCCA-College items. Due to the wide range of reading levels and heterogeneity of college student readers, MOCCA-College was developed to tap into this anomaly. Thus, The Flesch-Kincaid grade levels for the think-aloud and recall texts were within and at the lower range of MOCCA-College items (mean Grade level at 8.23 Flesch-Kincaid readability: 2 narratives = FK 7.5 and 8.4; 2 expository = FK 8.5 and 8.5).

**Table 2:** Text characteristics based on Coh-Metrix

	El Niño (E)	From Point A to B (E)	Sneaky Tactics (*N*)	Witnessing History (*N*)
Number of sentences (including title)	19	14	16	16
Word count	183	202	202	201
Flesch Reading Ease	62.97	66.98	75.07	66.17
Flesch-Kincaid Grade Level	6.93	7.50	5.93	7.15

*Note* E = expository, N = narrative

### Measures

**MOCCA-College.** The MOCCA-College is a self-paced maze task that includes 50 items with approximately half of the items being narrative and half being expository. Items were developed to address a wide range of readability levels (i.e., Grades 5.1–14.6 Flesch-Kincaid readability). Previous analyses have reported excellent internal consistency of the assessment based on inter-item correlations (Cronbach’s α = 0.92-0.95; Davison et al., 2020). The MOCCA-College is completed online using a secure link at the reader’s own pace with most readers needing about fifty minutes to complete the assessment in its entirety including instructions and practice items. MOCCA-College calculates four metrics for each participant: the number of responses indicating causally coherent inferences (i.e., CCIs, accurate response type), the number of responses indicating paraphrases (i.e., PARs, one of the inaccurate response types), the number of responses indicating elaborations (i.e., ELAs, one of the inaccurate response types), and the efficiency of correct responses (seconds per correct response). See Table 1 for descriptive statistics (maximum possible for MOCCA-College responses was 50).

**Nelson-Denny Reading Test.** To assess reading comprehension and reading rate, the Nelson-Denny Reading Test (NDRT) Form J subtest of reading comprehension was used. The NDRT is designed for the target population of the MOCCA-College (i.e., college students) and has good test-retest reliability (*r* =.94 for reading rate and *r* =.91 for comprehension) as well as good internal consistency (Cronbach’s a = 0.87; Fishco, 2019). There are seven text passages (both narrative and expository) in the reading comprehension subtext and a total of 38 multiple-choice questions. Each multiple-choice question has three possible options and one correct option. The text passages are present while students answer the multiple-choice questions. Participants are given 20 min to complete the subtest and to do their best to answer as many questions as they can in that time span. Forty-three of the participants (63%) did not finish the subtest in the allotted time. As part of the NDRT protocol, items that were not answered were marked incorrect. Reading rate is also determined during the first minute of the subtest. This is done by instructing participants to read at their typical rate and then record the number (indicating the word count) to the left of the reading line when the minute is complete. Descriptive statistics of index scores are in Table 1.

**Test of Word Reading Efficiency.** Decoding skills were assessed using two Test of Word Reading Efficiency-2 subtests (TOWRE-2; Torgesen et al., 2012), which have good test-retest reliability (Torgesen et al.). The Sight Word Efficiency subtest includes lists of real words and participants are instructed to read aloud as many as they can. The Phonemic Decoding Efficiency subtest includes lists of pseudowords, and participants are also instructed to read aloud as many as they can. In both subtests participants have 45 s to read as many words as possible. Descriptive statistics of scale scores are in Table 1.

## Procedures

Data collection sessions for the face-to-face validation study began with the experimenter greeting the participants and the participant providing informed consent. The first task was the think-aloud task in which the texts were presented by sentence on a computer screen. The experimenter instructed participants to read each sentence out loud and then state whatever thoughts they had following that sentence. After each text was read and thought aloud, participants were asked to recall that text and answer two yes-or-no questions about the text. A practice text was used in which the experimenter modeled the think-aloud process for the first half of the text and the participant thought aloud the remainder of the text (based on Fox et al., 2011; Hu & Gao, 2017). After the think-aloud and recall tasks, participants completed reading comprehension and reading rate measures (i.e., NDRT subtests) and decoding measures (i.e., TOWRE-2’s SWE and PDE; Torgesen et al., 2012) (see Authors, date, for more details and results from these measures). Finally, participants shared their demographic information and were debriefed about the study goals.

## Think-Aloud Coding

Oral think-aloud responses were recorded and later transcribed for coding. Participants thought aloud about each sentence of the texts. These verbal responses were then divided into idea units (i.e., expressions of complete thoughts such as a subject and a verb) and each idea unit was coded for the type of cognitive process used during reading. The think-aloud responses were coded by four researchers using a taxonomy based on previous research (van den Broek et al., 2001; 20% were scored in common with strong interrater reliability of κ = 0.85 [the interpretation of kappa between 0.80 and 0.90 being considered strong is based on McHugh, 2012] and any disagreements were discussed until codes were agreed upon). In this study, coded responses included different types of inferences, evaluations, paraphrases, text repetitions, and associations. Specifically, *valid elaborative inferences* included responses in which the participant explained the current sentence by referring to relevant background knowledge (e.g., “She must feel excited about going on a trip.”) or *predictive inferences* that used background knowledge to attempt to anticipate what would happen next or later in the text (e.g., “He will probably get caught for cheating at the card game.”). *Invalid elaborative inferences* included explanations of the text that were irrelevant, based on misconceptions in background knowledge, or were possibly due to an incorrect reading of the text (e.g., “Climate change will resolve itself.”). *Text-connecting inferences* included responses that connected back to previous text, either the text prior to the current sentence that was being thought aloud about or much earlier text. *Paraphrases* included responses that reworded the sentence just read and did not add any additional content or inferences based on background knowledge. *Text repetitions* included exact repetitions of the text just read. *Associations* included connections the participant made from the text to personal experiences (e.g., “That reminds me of when I drove through South Dakota.”).

### Recall Coding

The oral recalls of each text were also recorded and later transcribed for coding. The recalls were divided into idea units (i.e., expressions of complete thoughts such as a subject and a verb) and idea units were coded based on the part of the original text they corresponded to (κ = 0.93, interpreted as almost perfect per McMaster et al. 2014, interpretation that over 0.90 is almost perfect). The number of nonredundant idea units from the text were tallied to determine the total recall score (i.e., repetitions of the same idea unit were not included). Idea units were also coded as to whether the unit of text they corresponded to was highly connected in the cause-and-effect structure of the text or not. Following McMaster et al. (2012), idea units that had five or more causal connections within the text were coded as highly connected. A causal connection each time an idea unit was a cause or effect of another idea unit in the text.

## Results

To address our first research question regarding associations among the MOCCA-College responses and responses from the think-aloud and recall tasks, Kendell-tau correlations were conducted (Kendell-tau was chosen because some variables did not have normal distributions and Kendell-tau does not have assumptions of normality; Kendall & Gibbons, 1990; Van Doorn et al., 2018; see Table 3 for the correlation matrix).

**Table 3:** Kendell-tau correlations for MOCCA-C responses and efficiency measures

	Causally-coherent inference response (correct)	Elaboration responses (incorrect)	Paraphrase responses (incorrect)	Seconds/correct response (efficiency)
Reading Rate (NDRT)	0.10	− 0.04	− 0.10	− 0.30*
Comprehension (NDRT)	0.19*	− 0.15	− 0.17	− 0.25**
Sight Word Efficiency (TOWRE-2)	0.00	− 0.03	− 0.05	− 0.17
Phonemic Decoding Efficiency (TOWRE-2)	0.10	− 0.08	− 0.16	− 0.19*
Knowledge-based inferences (think aloud)	0.16	− 0.14	− 0.19	− 0.13
Text-connecting inferences (think aloud)	0.01	0.03	− 0.01	− 0.09
Paraphrases (think aloud)	− 0.04	0.06	0.01	0.01
Elaborations (think aloud)	− 0.29**	0.22*	0.27**	− 0.06
Total idea units recalled	0.25**	− 0.21*	− 0.22*	− 0.14
Highly-connected idea units recalled	0.11	− 0.05	− 0.12	− 0.25**
Other idea units recalled	0.28**	− 0.24**	− 0.22**	− 0.09

Note: *N* = 63. Higher values of seconds per correct response are indicative of slower reading times and less efficient reading

First, to examine construct validity with regards to how MOCCA-College items were associated with the think-aloud responses, we found that the CCI responses were not reliably positively associated with valid elaborative inferences in the think-aloud responses as expected; however, they were negatively correlated with the invalid elaborative inferences. The negative association between the CCI responses and invalid elaborative inferences can help support the construct validity for how the CCI responses were developed. As such, the CCI response type was developed to close the causal gap when completing MOCCA items. Thus, because this finding points to a negative association between the MOCCA-College CCIs and invalid elaborative inferences from a think-aloud task that do not connect to relevant and causal background knowledge, this finding partially supported our development goals for this assessment.

Second, MOCCA-College ELA and PAR responses were both reliably positively correlated with the invalid elaborative inferences from the think-aloud responses. This finding also partially supported the MOCCA-College development and were based on prior research (i.e., Carlson et al., 2014; McMaster et al., 2012). For instance, the association between the ELAs and invalid elaborative inferences from the think-aloud task supported the development of the ELA response types, of which were developed based on findings from prior research with children to identify struggling comprehenders’ generation of connections to background knowledge not necessarily relevant with the text. However, the PAR responses were developed to identify another group of struggling comprehenders’ who have been shown to paraphrase the goal, subgoal, or main idea statements in text. The PAR responses in a MOCCA-College item generally include a paraphrase of information from the second or third sentence of the text, or a combination of the two, which can include the goal/subgoals or main idea of the text and require the reader to connect back in the text and generate the paraphrase (i.e., or pick the PAR response). A positive correlation between the MOCCA-College PARs and invalid elaborative inferences from the think-aloud task shifted from how this response type was developed; however, could point to the need to reconceptualize the adult/college struggling comprehender and types of processes they may be generating during reading (e.g., perhaps more inferences versus paraphrases).

To address the final component of our first research question, regarding construct validity for how MOCCA-College items are associated with recall responses, we found that the CCI responses positively correlated with total idea units recalled, as well as idea units that were not highly connected to the causal structure of the text (i.e., these were responses coded as ‘other idea units recalled’). This finding was unexpected since previous research identified that generating causal inferences (i.e., CCI responses) during reading (i.e., the process) leads to the development of a causally coherent situation model after reading (i.e., the product) (e.g., Trabasso & van den Broek, 1985). Additionally, we found that MOCCA-College ELA and PAR responses were negatively correlated with the total idea units recalled and other idea units recalled. These findings were also unexpected for this analysis; however, need to be further explored. Prior research has found that struggling adolescent readers have difficulty developing coherent representations of text after reading due to generating inefficient cognitive processes during reading (McMaster et al., 2012; Rapp et al., 2007). Thus, this finding, although not expected, supports this notion, and extends this finding to college students. However, given this finding was not associated with our expectations for this study, additional research would need to be conducted to support this claim.

To address our second research question, and to further support the findings from our first research question, we controlled for decoding skills because think-aloud responses may co-vary and used generalized linear models and examined associations between the think-aloud responses and MOCCA-College responses (see Table 4). This analysis allowed for an understanding of how cognitive processes expressed in think-aloud responses uniquely predicted MOCCA-College responses and efficiency scores while accounting for decoding skills. Models were estimated using the glm2 package with R software as this package allowed for robust analyses with nonnormal variable distributions (Fox, 2015; Marschner, 2011). In each of the models, valid elaborative inferences (i.e., for this analysis, elaborative and predictive inferences that connect to valid background knowledge associated with the text were combined), text-connecting inferences (i.e., connections across ideas in the text), invalid elaborative inferences (i.e., for this analysis, elaborative and predictive inferences that connect to invalid background knowledge associated with the text and associations to general experiences were combined), and paraphrases (i.e., for this analysis paraphrases and text repetitions were combined) were entered as predictors (N.B.: sight word efficiency and phonemic decoding efficiency scale scores were also considered as predictors, but were not statistically significant. These decoding measures were not included in the reported models out of concerns for parsimony).

**Table 4:** Results of regression models

Generalized linear model of think aloud responses predicting MOCCA-College CCI responses (correct)				
	β	SE	t	*p*
Valid EL inferences (think aloud)	0.02	0.01	2.67	0.01
TC inferences (think aloud)	− 0.02	0.01	-1.07	0.28
Paraphrases (think aloud)	0.01	0.01	1.18	0.24
Invalid EL inferences (think aloud)	− 0.08	0.02	-5.05	< 0.001

Generalized linear model of think aloud responses predicting MOCCA-College PAR Responses (incorrect)				
	β	SE	t	*p*
Valid EL inferences (think aloud)	− 0.08	0.02	-4.75	< 0.001
TC inferences (think aloud)	0.15	0.05	1.71	0.09
Paraphrases (think aloud)	− 0.02	0.03	-2.34	0.02
Invalid EL inferences (think aloud)	0.17	0.04	7.36	< 0.001

Generalized linear model of think aloud responses predicting MOCCA-College ELA Responses (incorrect)				
	β	SE	t	*p*
Valid EL inferences (think aloud)	− 0.07	0.02	-4.39	< 0.001
TC inferences (think aloud)	0.10	0.05	2.66	0.01
Paraphrases (think aloud)	− 0.03	0.03	-1.07	0.29
Invalid EL inferences (think aloud)	0.22	0.04	3.45	< 0.001

Generalized linear model of think aloud responses predicting seconds per correct item on MOCCA-College (efficiency)				
	β	SE	t	*p*
Valid EL inferences (think aloud)	0.00	0.00	1.17	0.25
TC inferences (think aloud)	0.00	0.00	1.03	0.31
Paraphrases (think aloud)	0.00	0.00	-0.01	0.82
Invalid EL inferences (think aloud)	0.00	0.00	0.23	0.82

Notes *N* = 63. β = standardized beta coefficient, *t* = *t*-test value, *p* = significance. Valid EL inferences = number of elaborative and predictive think-aloud responses that were valid knowledge-based inferences, TC inferences = number of think-aloud responses that were text-connecting inferences, Paraphrases = number of think aloud responses that were text repetitions or paraphrases, Invalid EL inferences = number of think aloud responses that were invalid elaborative or predictive inferences or associations

In the first model, the number of MOCCA-College CCI responses were used as the dependent variable (in Models 2–4, the dependent variable was MOCCA-College PARs, ELAs, and efficiency score respectively). As can be seen in Table 4, valid elaborative inferences from the think-aloud task positively predicted CCI responses, and invalid elaborative inferences from the think-aloud task negatively predicted CCI responses. When MOCCA-PAR responses were used as the dependent variable, valid elaborative inferences from the think-aloud task negatively predicted PAR responses, and invalid elaborative inferences from the think-aloud task positively predicted PAR responses. When MOCCA-ELA responses were used as the dependent variable, valid elaborative inferences from the think-aloud task negatively predicted ELA responses, text-connecting inference positively predicted ELA responses, and invalid elaborative inferences from the think-aloud task positively predicted ELA responses. Finally, when the seconds per item on MOCCA-College (i.e., MOCCA-College efficiency) were used as the dependent variable, none of the think-aloud responses were significant predictors of average seconds per correct item. Using more robust analyses than the correlations, these findings help further support the findings from the first research question, as well as the goals for the developed MOCCA-College response types.

Finally, to address our third research question regarding whether MOCCA-College responses correlated with previously established reading measures as evidence of criterion validity, we found that seconds per correct response (MOCCA-College efficiency metric) negatively correlated with the NDRT reading rate and comprehension scores as well as the phonemic decoding efficiency measures on the TOWRE-2 (note that more seconds per correct item indicates less efficiency). When looking only at the MOCCA-College CCI responses, we found that the CCI responses were positively correlated with the NDRT comprehension subtest scores only. These findings supported our expectations for establishing criterion validity between MOCCA-College and other reading measures.

## Discussion

The purpose of this study was to examine initial construct validity and criterion validity for the MOCCA-College, which is a novel diagnostic reading comprehension assessment for college students. Specifically, we examined the construct validity of MOCCA-College through general and predictive relationships between the MOCCA-College response types and efficiency scores and responses from think-aloud and recall tasks. In addition, we examined the criterion validity of MOCCA-College through general relationships between MOCCA-College components and previously developed reading measures. Overall, the findings from this study support the development of MOCCA-College items and their associated response types based on relationships found between similar methods and assessments of reading and for identifying reading comprehension processing skills.

### Construct Validity of MOCCA-College

#### Relationships Between MOCCA-College Response Types and Think-Aloud Responses

Based on the think-aloud evidence to assess construct validity, when examining correlations, correct responses (i.e., CCIs) on the MOCCA-College were negatively associated with the invalid elaborative inferences from the think-aloud task. These invalid elaborative inferences from the think-aloud task were coded as such when readers were identified as generating connections from the text to background knowledge but to knowledge that may not help develop causal coherence during reading. This finding both supports previous findings in the field, as well as the development of MOCCA-College. When developing MOCCA-College, the CCI responses were developed based on prior research that has shown how readers generate causal inferences during reading to help close gaps in the text that are not fully explained (Trabasso & van den Broek, 1985). Given that we found a negative association between choosing the CCI response type and readers generating invalid elaborative inferences during their think alouds, this partially supports initial content validity between our development goals for the CCIs on MOCCA-College.

Additionally, to support the correlations between the MOCCA-College response types and the think-aloud responses, we further assessed the predictive nature of these data. Although the CCIs were not positively correlated with the valid elaborative inferences from the think-aloud task, we did find that valid elaborative inferences positively predicted CCI responses on MOCCA-College, and invalid elaborative inferences negatively predicted CCI responses on MOCCA-College. These findings confirm our expectations regarding content validity between the development of MOCCA-College response types and other tasks that identify similar reading comprehension processes. However, because we had mixed findings between the valid elaborative inferences and the CCI responses on MOCCA-College, additional examination of the CCIs is warranted. For instance, we may need to consider the difficulty level of these response types, and perhaps, overall, for the MOCCA-College items. MOCCA-College items were developed with a large range of readability levels to explore college readers’ comprehension difficulties. This may have inadvertently created a misrepresentation of the data; thus, resulting in too many items that were too easy. Future research should focus on examining potential associations between the more difficult MOCCA-College items and their corresponding CCI response types and readers’ think-aloud responses.

Furthermore, incorrect response types on the MOCCA-College corresponded with using background knowledge in manners that do not support coherence. First, we found that the MOCCA-College ELA responses were positively correlated with the invalid elaborative inferences from the think-aloud task and positively predicted MOCCA-College ELA responses, as well as valid elaborative inferences from the think-aloud task negatively predicted MOCCA-College ELA responses. These findings support the goals of developing the ELAs for MOCCA-College which are based on prior think-aloud research. That is, MOCCA-College ELAs were developed and based on previous think-aloud findings that have shown how one group of struggling comprehenders tends to connect to their background knowledge during reading, but the connections are often at the expense of generating causal inferences and instead lead the towards irrelevant prior knowledge connections or general associations tangentially associated with their experience. These prior findings have been found with some adult/college samples of readers (e.g., Magliano et al., 2011); however, the majority of this research, and specifically the identification of subgroups of struggling comprehenders have been found with readers in late elementary grades (Carlson et al., 2014; Karlsson et al., 2018; McMaster et al., 2012). Thus, this finding supports our development efforts, as well as extends previous research conducted to identify profiles or subgroups of struggling comprehenders with adult/college readers. The development of MOCCA-College could help validate the notion that subgroups of struggling comprehenders exist, even at the college level.

One other subgroup of struggling comprehenders found in previous research with children is a group of readers who tend to paraphrase more often during their think alouds than generate other processes (Carlson et al., 2014; Karlsson et al., 2018; McMaster et al., 2012). The MOCCA-College PARs were developed with this subgroup of struggling comprehenders in mind. Findings from the current study to evaluate the association between the MOCCA-College PARs and paraphrases generated during the think-aloud task was not supported. Instead, we found a positive correlation between the MOCCA-College PARs and the invalid elaborative inferences from the think-aloud task, as well as the paraphrases from the think-aloud task negatively predicting MOCCA-College PAR responses and text-connecting inferences during the think-aloud task positively predicting MOCCA-College PAR responses. These findings, although not what we expected, are interesting. First, perhaps these findings lend themselves to first consider the difficulty levels again of MOCCA-College items and the corresponding response types, and second, that if subgroups of struggling comprehenders do exist, they may be developmentally different at the college level compared to late elementary readers. For instance, paraphrases may be less commonly generated during reading at this age, but instead, inferences like text connecting and elaborative inferences are more likely to be generated. Prior research has found that assessments that resemble both human coding think-aloud protocols and MOCCA-College positively predict that college readers’ not identified as necessarily struggling, successfully able to generate elaborative and global text-connecting inferences during reading comprehension (Magliano et al., 2011). However, these conclusions are speculative and would need additional research to appropriately support them, of which we intend to explore additional development options for MOCCA-College items.

### Relationships Between MOCCA-College Response Types and Recall

The MOCCA-College was also examined in terms of its associations with memory for the texts via the recall task. Overall recall was positively correlated with the MOCCA-College CCI response types (i.e., accuracy), but this correlation was constrained to idea units that were not highly connected with a causal structure of the text. Previous research has found that in general, highly connected ideas are more likely to be recalled than other ideas in the text because of their causal structure (van den Broek, 1994). Moreover, readers in college typically have sufficient skills that are sensitive to the structural centrality of the text, meaning they would recall highly connected idea units (van den Broek & Helder, 2017). Therefore, one possible explanation for the findings in the current study is that readers across coherence skill levels recalled the highly connected units, but remembering other idea units required more skill and could not be teased apart with the coding of this recall task. This could also explain why MOCCA-College efficiency scores (seconds per correct response) were negatively correlated with the highly connected idea units from the recall task. However, the difference in findings between MOCCA-College CCIs and efficiency scores were not anticipated given that previous work on sensitivity to structural centrality and comprehension skill has not differentiated whether skill was measured in terms of accuracy or efficiency (e.g., van den Broek & Helder). However, determining the correct response on the MOCCA-College efficiently requires quickly identifying the main point or goal of the item, which would be a highly connected idea central to the text. Overall accuracy is not dependent on time and may indicate more thoughtfully processing the text to notice details that are not highly connected. This is merely conjecture and not supported by empirical evidence but would explain why the recall findings were different for accuracy and efficiency.

### Criterion Validity of MOCCA-College

The findings associated with identifying criterion validity for the current study are different from previous findings on an elementary school version of MOCCA (Carlson et al., 2014). The efficiency metric of the MOCCA-College (seconds per correct item) correlated with established measures of reading rate, comprehension, and decoding which supports the criterion validity. This is important due to there being limited assessments in the field to corroborate reading comprehension efficiency, especially for college readers. Moreover, the number of correct responses on the MOCCA-College positively correlated with the NDRT reading comprehension scores which was evidence of criterion validity. Phonemic decoding was significantly correlated with the efficiency measure, but not with overall accuracy. This finding may be explained by the fluency of the readers in conjunction with their ability to generate necessary processes (e.g., inferences). This explanation is consistent with evidence that reading efficiency better explains general reading skills than do either accuracy or speed (reading time without considering accuracy; Ciancio et al., 2015; Hale et al., 2012). In other words, efficiency may be more of a general measure of reading skill than accuracy. This is likely because to reading efficiently requires automaticity of skills to afford the cognitive capacity to read both quickly and accurately (Samuels & Flor, 1997). This would explain why interventions that facilitate automaticity in reading benefit efficiency more than accuracy (Blonder et al., 2019; Therrien, 2004).

NDRT comprehension scores only had a small correlation with MOCCA-College accuracy and efficiency. Because both the NDRT comprehension subtest and the MOCCA-College are designed to assess comprehension, it was expected that scores on these measures would positively correlate. The small size of the correlation could likely be due to the NDRT being time limited and the MOCCA-College being self-paced. 63% of the students in this study were unable to complete the NDRT in the time allotted and missed items were marked as incorrect. Therefore, an incorrect answer may have not indicated an inaccurate understanding of the text rather a lack of time to complete the assessment. However, due to the protocol for scoring NDRT, incomplete answers are scored as incorrect. In contrast, students were only eligible for this study if they completed all of the items on the MOCCA-College. This proposed explanation is consistent with previous findings that timed and untimed measures of reading skill do not strongly correlate with each other (Goldhammer et al., 2021).

### Taking These Findings Together

Overall, the findings from this study extend prior research by providing a valid new tool for identifying how readers process text during reading. One of the most typical methods used to assess readers’ cognitive processes generated during reading comprehension is the think-aloud method. This method has provided a host of informative information to the field regarding how readers process text. However, the think-aloud method is quite laborious to use in research due to the time it takes to collect and code the data for interpretation. MOCCA-College could, thus, be used to obtain similar data with more efficiency.

The MOCCA-College is designed to assess college readers’ skills in making appropriate inferences needed to develop a coherent mental representation of a text integrating background knowledge appropriately (i.e., a situation model). Based on the findings of the think-aloud task in this study, accuracy on the MOCCA-College (i.e., CCI response score) does measure what it intends to measure regarding inferences. We found a positive association between accurate responses on the MOCCA-College and knowledge-based inferences. These inferences are essential for constructing accurate situation models (Bos et al., 2016; Graesser et al., 1994, 1997; Kintsch, 1998). Furthermore, we found a negative association between accurate responses on the MOCCA-College and elaborations (invalid inferences and associations). Unlike valid inferences, invalid inferences and associations appear to lead to incoherent mental representations of the text and inaccurate situation models (Diakidoy et al., 2011, 2016; Kendeou & van den Broek, 2005; Kendeou & Broek, 2007).

As mentioned, the incorrect response types on the MOCCA-College were developed to relate to different comprehension processes that struggling readers engage in based on prior think-aloud research findings: paraphrases and elaborations. However, the think-aloud findings were similar for both response types; that is, negative associations with knowledge-based inferences and positive associations with elaborations. Unlike the MOCCA for elementary school students (Carlson et al., 2014), there was no association between the PAR response types on the MOCCA-College and paraphrases in the think-alouds. Furthermore, there was no indication of a negative association between the CCIs on the MOCCA-College and paraphrases in the think-alouds. These null findings may be due to the different uses of paraphrases by readers. Paraphrases may serve a variety of functions, it may be used as memory device (Coté et al., 1998) or as an initial step to better understand the text so that inferences could be made (McNamara, 2004). Paraphrases have also been found to be produced more when readers have a goal of studying compared to a goal of entertainment (Bohn-Gettler & Kendeou, 2014; Linderholm & van den Broek, 2002; van den Broek et al., 2001), which provides evidence that paraphrases may be used along with inferences to build coherence. However, paraphrasing does not require making connections in the text and subsequently may not assist in building and maintaining coherence (Magliano & Millis, 2003). Readers may opt to use paraphrases instead of inferences and subsequently paraphrases lead to a less well-developed situation model (McMaster et al., 2012).

These different uses of paraphrases (either to facilitate situation model development or in lieu of coherence building processes) may explain the lack of association between paraphrases and CCI as well as the PAR response types on the MOCCA-College. In some situations, there may have been coherence building benefits of paraphrases in the think-aloud response, but in others they were used instead more effective methods of building and maintaining coherence (i.e., inferences). Therefore, it is possible that the combination of these two functions of paraphrases lead to null results. The lack of significant associations with paraphrases in the think-aloud responses and the other reading measures in this study support this possibility. However, without knowledge of how the paraphrases specifically operated for the readers in this study, it is challenging to make conclusions.

### Limitations and Future Directions

Although the findings of the current study support initial evidence of content and criterion validity, there are limitations that need to be acknowledged. First, the sample overrepresented students who identified as women and White. Given evidence that inference skills may vary by gender in both children and adults (Clinton et al., 2014; Hannon, 2014), the gender imbalance is concerning. Moreover, the demographics of college students are shifting to have fewer White students and more students from minoritized racial and ethnic backgrounds (Payne et al., 2017). Therefore, future studies on reading comprehension in college students should address how to have more diverse representation in participant samples.

Second, the sample size for this study was not large. This is typically the case with think-aloud studies because the methodology is time and labor intensive (e.g., Bohn-Gettler & McCrudden, 2018; Clinton & van den Broek, 2012; Kendeou et al., 2019; Máñez et al., 2019; Yeari & Lavie, 2021). Furthermore, the sample for this the think-aloud portion of this study may have not been representative of the struggling college reader, which is also reflected in their NDRT scores. Future research should aim to target readers specifically struggling with comprehension processing. However, recruitment of this population is challenging given that struggling readers may tend to shy away from reading comprehension studies. Thus, a more concerted effort of recruitment with educators and supporting struggling college readers’ needs for these types of studies is warranted.

Third, the texts used in the think-aloud task, as well as some of the items in MOCCA-College may have been too easy for the targeted population of readers. The goals of using easier texts for the think-aloud task in terms of readability, and of developing MOCCA-College items at lower grade levels, were to eliminate any issues with decoding and target reading comprehension processing difficulties. Future MOCCA-College refinement and development studies may, thus, focus on using more difficult texts to support both readability and decoding skills, as well as continue to examine reading comprehension processing skills.

Finally, in terms of validity, the lack of association between paraphrases in think-aloud responses and the PAR response type in the MOCCA-College is concerning. Given that the MOCCA with elementary school readers did have such an association, it is possible that paraphrases function differently for this age group. Another possibility, of course, is that the PAR response type on the MOCCA-College did not adequately capture this cognitive process while reading, or the development of an additional response type such as text-connecting inferences may be worth investigating further. Based on these findings and other evidence on item performance, the MOCCA-College is undergoing refinement and further validation.

## Conclusion

In summary, the MOCCA-College is a promising assessment of identifying coherence-building skills in college readers. Accuracy (i.e., CCIs) on the MOCCA-College indicated that college readers engage in coherent-building connections to background knowledge and inaccuracy (i.e., PARs, ELAs) indicates these readers make inappropriate and inaccurate connections to background knowledge that do not build coherence. Furthermore, efficiency on the MOCCA-College was indicative of automaticity in reading skills. This study provides preliminary evidence that the MOCCA-College may be a valid and useful measure of college students’ reading comprehension processing skills and could be used with supplemental programs to help guide students on how to improve their reading comprehension processing skills. Furthermore, MOCCA-College has the capacity to provide both the educator and the individual student feedback on the scores and guidance for how to interpret the scores. Findings from the current study support the need to bring MOCCA-College to settings to help guide college students’ reading comprehension needs.

## Footnotes

1 MOCCA is an acronym for Multiple-choice Online Causal Comprehension Assessment and is now only referred to as MOCCA through copyright processes.
